# Study and Optimization of a Micro-Structured Waveguiding and Fluorescent Sol-Gel Architecture

**DOI:** 10.3390/molecules28124608

**Published:** 2023-06-07

**Authors:** Ibtihel Marzouk, David Riassetto, Alain Morand, Davide Bucci, Michel Langlet

**Affiliations:** 1 LMGP, Grenoble INP, CNRS, University Grenoble Alpes, F-38000 Grenoble, France; ibtihel.marzouk@grenoble-inp.fr (I.M.); david.riassetto@grenoble-inp.fr (D.R.); 2 IMEP-LAHC, Grenoble INP, CNRS, University Grenoble Alpes, F-38000 Grenoble, France; alain.morand@grenoble-inp.fr (A.M.); davide.bucci@phelma.grenoble-inp.fr (D.B.)

**Keywords:** sol-gel processing, diffraction grating, channel waveguide, fluorescence intensity

## Abstract

Channel waveguides with diffraction gratings at their input and output for light injection and extraction, respectively, constitute the key components for applications in integrated optics and photonics. Here, we report for the first time on such fluorescent micro-structured architecture entirely elaborated on glass by sol-gel processing. This architecture particularly takes advantage of a high-refractive index and transparent titanium oxide-based, sol-gel photoresist that can be imprinted through a single photolithography step. This resist enabled us to photo-imprint the input and output gratings on a photo-imprinted channel waveguide doped with a ruthenium complex fluorophore (Rudpp). In this paper, the elaboration conditions and optical characterizations of derived architectures are presented and discussed with respect to optical simulations. We firstly show how the optimization of a two-step deposition/insolation sol-gel procedure leads to reproducible and uniform grating/waveguide architectures elaborated on rather large dimensions. Then, we show how this reproducibility and uniformity govern the reliability of fluorescence measurements in waveguiding configuration. These measurements demonstrate that: (i) our sol-gel architecture is well adapted to the efficient channel–waveguide/diffraction grating coupling at the Rudpp excitation and emission wavelengths; (ii) it enables an efficient propagation of the emission signal in the core of the waveguide allowing its photo-detection after extraction through the output grating; and (iii) it is affected by very reduced parasitic mechanisms, such as propagation losses and photobleaching features. This work constitutes a promising preliminary step toward the integration of our architecture in a microfluidic platform for further fluorescence measurements in liquid medium and waveguiding configuration.

## 1. Introduction

Miniaturized components in the form of integrated channel waveguides have been largely exploited in optics and photonics. These structures are based on the optical power field confinement in the lateral and vertical directions, and allow light propagation by total internal reflection along the axial core of the waveguide, according to a suitable mismatch between its refractive index and those of the substrate and superstrate. Over the two past decades, such integrated waveguides have, for instance, been studied as optical sensors in biological and bio-medical applications. The presence of a target analyte in the surrounding medium induces changes in optical properties (absorption coefficient, refractive index, fluorescence intensity, plasmon resonance, etc.), at the waveguide surface. This, in turn, modifies the signal propagating along the waveguide core. These modifications can then be measured with traditional sensitive photon detection techniques, such as photomultiplier tubes and charge-coupled devices. Derived optical biosensors are integrated in microfluidic platforms adapted to measurements in liquid medium, which results in the fabrication of miniaturized and portable sensors. Such sensors have been proposed to probe fluorescently labelled biomolecules [1,2] or for label-free, bio-detection applications [3,4]. They have also been exploited for the in situ measurement of physical–chemical parameters, such as dissolved oxygen concentration or pH that are two essential parameters for monitoring the viability of biological cells in aqueous medium [5,6]. These works depict the widespread range of probed analytes and sensing principles that can be considered using optical waveguides intended to biological and bio-medical applications. They also highlight that, because of light confinement in the core of the waveguide, the increased light-analyte interaction results in enhanced sensitivity and fast response, which offers an optimal solution for real-time and on-site detection. As demonstrated in several lab-on-chip reports, among the numerous techniques used, the sol-gel process has been shown to be a suitable route for the elaboration of channel waveguide optical and photonic sensors [7,8,9], and some papers report on the sol-gel processing of channel waveguide-based integrated biosensors [1,5]. These works demonstrate that the sol-gel process provides an ideal trade-off between low cost and easy implementation, optical quality, as well as mechanical and chemical robustness, and it can be conveniently implemented without requiring a clean room.

The main issue in the exploitation of channel waveguides relies on the achievement of an efficient light coupling with the waveguide core, and this issue appears all the more challenging for thin waveguides. From that point of view, the traditional end-fire butting method involving coupling with an optical fiber is rather demanding since it necessitates a rigorous end facet polishing of the waveguide and, in the case of thin waveguides, it requires a very critical fiber-waveguide alignment tolerance. Several alternative approaches have thus been proposed, such as micro-lensed fibers [10] or different variants of taper couplers [11,12,13]. However, these options still suppose waveguide (or taper) facet polishing and they do not solve coupling issues with fibers, whose diameter remains much greater than the height (250 nm) of waveguides considered in this work, even for micro-lenses of very small mode-field diameter (~2 µm for standard micro-lensed fibers). Thus, we have chosen another alternative based on light coupling with diffraction gratings. Such a coupling requires a sufficiently high-refractive index periodic structure composed of fine patterns whose period should ideally be in the order of the considered wavelength. It allows relaxed positioning tolerances, does not require waveguide facet polishing, and is fully compatible with device miniaturization [14,15]. Furthermore, it is well adapted for integration in a microfluidic platform intended to light measurements in a liquid medium, and it has already been exploited in optical biosensor applications [3,16,17,18]. However, the method has only been the object of punctual studies in this field and its potential has not been sufficiently examined. Besides, despite the well-recognized potential of sol-gel derived integrated devices, only very rare papers report on the sol-gel fabrication of integrated waveguides endowed with grating couplers. For instance, Enami reported on such sol-gel derived gratings elaborated with a nanoimprint method and he mentioned the difficulty to fabricate gratings on large areas [19]. This lack in sol-gel devoted works and derived performances shows that the presently available knowhow in sol-gel processing is not sufficiently adapted yet, which introduces the objectives and originality of the work presented here.

We have recently studied a new sol-gel procedure that can strongly enhance the necessary knowhow [20,21]. We particularly took advantage of a transparent and high-refractive index, titanium–oxide (TiO_2_)-based, sol-gel photoresist that can be imprinted through a single insolation/development photolithography step, thus avoiding multi-step and rather costly traditional lithographic procedures. This resist enabled us to propose a specific protocol leading to micro-structured architectures composed of diffraction gratings imprinted on channel waveguides. Furthermore, in order to assess the ability of diffraction gratings to in-couple (out-couple) light in (from) the channel waveguides, the latter have also been doped with the fluorophore tris(4,7-diphenyl-1,10-phenanthroline)ruthenium(II) (Rudpp). This metal–organic complex exhibits large excitation and emission bands around 460 and 610 nm, respectively, and the fluorescence emission of Rudpp is selectively quenched in the presence of oxygen, leading to an emission intensity or lifetime decrease when increasing molecular–oxygen concentration. Accordingly, Rudpp is one of the most widely studied complexes for dissolved oxygen sensing by fluorescence quenching in the real-time control and monitoring of cell cultures [5], and it has been very often proposed for the sol-gel processing of such sensors in waveguiding nor configuration [5,22,23]. As we are ultimately interested in such application, Rudpp is the model fluorophore that we chose in this work to assess our architecture. However, studies devoted to Rudpp generally rely on its encapsulation in an organic polymer or silica-based host matrix. The low-refractive index of such matrices hardly fulfills light-guiding requirements, i.e., a suitable index mismatch with the substrate and superstrate, and it is also expected to reduce the diffracting efficiency of light couplers imprinted on the waveguides. The high index of a TiO_2_-based matrix allows to fulfill these requirements and such a sol-gel-derived matrix has already been proposed for the encapsulation of a Ru complex fluorophore [24]. However, the micro-structuration of a TiO_2_ sol-gel matrix doped with Rudpp has never been proposed for the fabrication of channel waveguide/diffracting-coupler architectures. We previously showed that the proposed resist is compatible with Rudpp encapsulation and oxygen sensitivity, i.e., with the diffusion of molecular oxygen within the resist thickness enabling contact with the encapsulated Rudpp molecules and activating their photo-response toward oxygen [20]. We also demonstrated the feasibility of a sol-gel-derived, micro-structured channel waveguide/diffracting grating architecture arising from the localized photo-imprinting of our sol-gel derived, TiO_2_-based resist [20,21]. A simplified architecture on glass composed of channel waveguides endowed with diffraction gratings at only one of their ends, has thus been the object of first assessments showing a large tolerance in the angular and horizontal/vertical positioning of the optical fiber used for light injection in the waveguide core, which confirmed all the potential of a diffraction grating coupling [21]. However, the obtained diffraction gratings still suffered from a lack of uniformity and reproducibility in their dimensionalities (height, width and periodicity), which hindered further accurate assessment of our waveguiding architecture potential. 

In the present paper, we describe for the first time investigations performed on a complete sol-gel architecture on glass composed of Rudpp-doped channel waveguides endowed with diffraction gratings at their input and output. This experimental work was also supported by optical simulations. In the first part of the paper, we showed how the sol-gel protocol based on a TiO_2_-based photoresist had been optimized, yielding uniform and reproducible gratings imprinted on the waveguides. Then, this optimized architecture enabled us to perform accurate assessments based on fluorescence measurements in a configuration adapted to an integration in a microfluidic platform, which are described in a second part of the paper. These measurements demonstrate that our architecture allows reproducible in-coupling of the excitation signal in the waveguide core, guided propagation of the excitation and emission signals, and out-coupling of the emission signal toward a photo-detector. To summarize, all the novelty of this work relies on the optimization and assessment of an original, double-grating/channel waveguide, fluorescent architecture. This one has been entirely elaborated by sol-gel processing, which has never been proposed before in the literature. Furthermore, this architecture takes advantage of an original TiO_2_-based sol-gel photoresist that can be imprinted through a single insolation/development photolithography. As demonstrated in this paper, this photoresist leads to diffraction gratings imprinted on rather large dimensions, which constitutes a major innovation compared to the sol-gel state of the art. These studies constitute an essential first step toward the ultimate integration of our architecture in a microfluidic platform for fluorescence measurements in liquid medium and waveguiding configuration.

## 2. Results and Discussion

### 2.1. Optical Simulations

**Figure 1:**
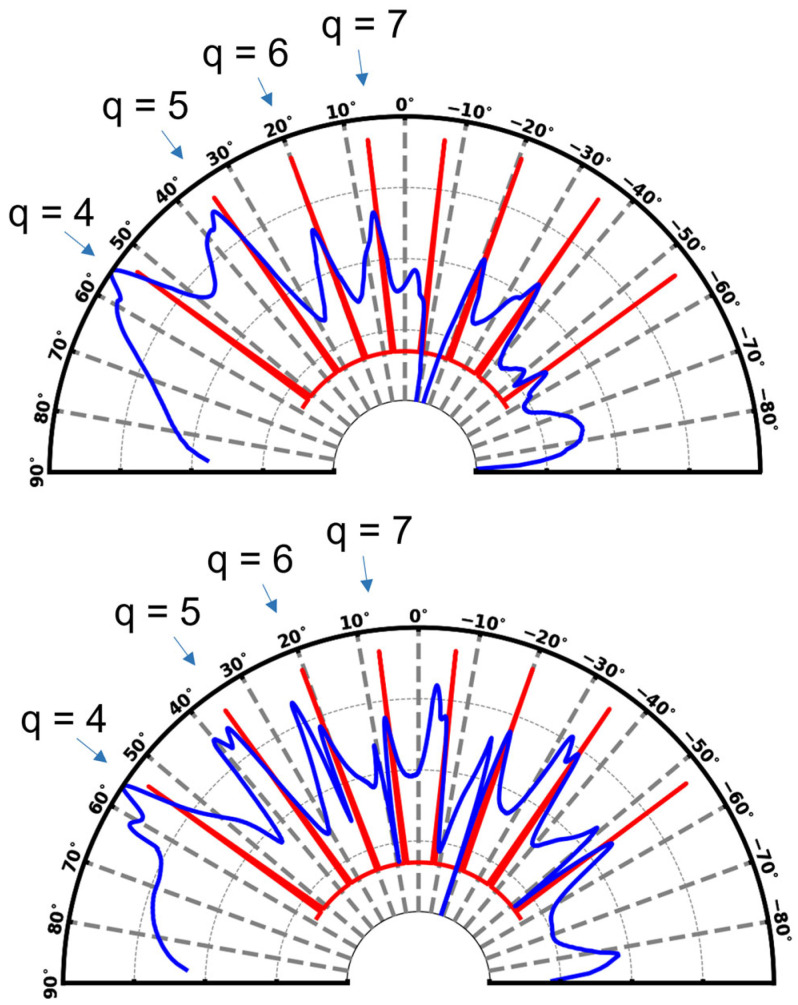
Simulated angular dependance of the intensity profile for light injected at λ = 460 nm in a channel waveguide through a diffraction grating with air (**top**) and water (**bottom**) as a superstrate medium. The light intensity has been normalized according to the maximal value obtained. The red lines depict the theoretical diffraction angles deduced from the grating law (Equation (1) in Section 3).

The angular dependance of channel waveguide/diffraction grating light coupling was simulated by RCWA for gratings with a trapezoidal profile (see next sub-section) and a mean width/pitch of 1 µm/2 µm, imprinted on channel waveguides with a width/height of 200 µm/250 nm. It is illustrated in Figure 1 (top) at the Rudpp excitation wavelength (460 nm) with air as a superstrate medium. This figure shows some series of coupling peaks that depict light diffraction through the grating according to a finite number of injection angles. The illustrated peaks exhibit a certain broadness and are positioned at angle values slightly shifted compared to those predicted by the grating law (also illustrated in Figure 1 for comparison purpose). These differences arise from the influence of grating dimensionalities that are not taken into account by the grating law. The main peaks are located around 55°, 35°, 20° and 8° vs. the vertical direction, and are associated with coupling orders q = 4, 5, 6 and 7, respectively. The most intense peak is located around 55°, which corresponds to an optimal coupling of the excitation signal with the channel waveguide. Figure 1 also illustrates splitting off the different coupling peaks, which is depicted by a shoulder on the high-angle side of the peaks. It is probably due to the fact that the channel waveguide below the grating supports two propagation modes. Each mode would be explained by two different light pathways governed by the incidence angle and that fulfill total internal-reflection conditions, and the associated diffracted power would, in turn, depend on the diffracted angle according to the grating profile. 

As our micro-structured architecture is ultimately intended for further exploitation in water, similar simulations were also performed by considering water as a superstrate medium (Figure 1, bottom). Interestingly, and despite the refractive index difference between air (*n* = 1) and water (*n* = 1.33), we observe similar behaviors for both considered media with an optimal excitation-signal coupling for an incidence angle around 55°. The main difference between both media is a more marked splitting of the coupling peaks in water. At the emission wavelength (610 nm), optical simulations showed that the main coupling peaks appeared located around 44°, 21° and 2° (not illustrated here), which were associated with coupling orders q = 3, 4 and 5, respectively. These simulations also indicated that light coupling depends on the diffraction grating height and length (number of illuminated grating periods). More specifically, in the reduced range of grating heights considered here, they showed that, while the optimal coupling angle is not significantly influenced, the efficiency of light coupling is enhanced when the grating height increases from ~160 nm to ~250 nm (not illustrated here). These simulation results were exploited to further optimize our experimental conditions (grating elaboration and fluorescence measurements in guided configuration), as presented and discussed in the upcoming sections. 

### 2.2. Channel Waveguide/Diffraction Grating Architecture Elaboration

#### 2.2.1. Optimization of the Experimental Protocol

**Figure 2:**
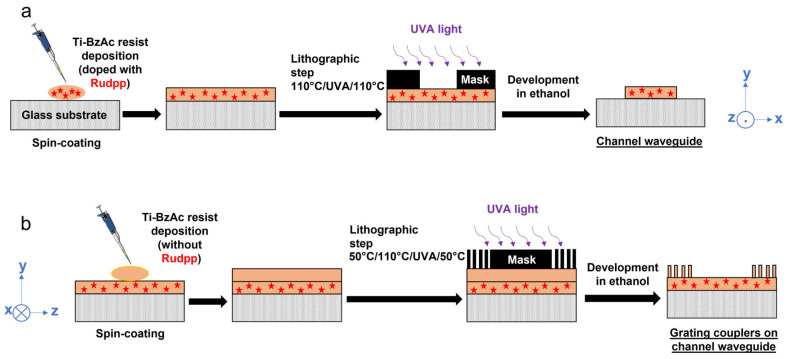
Schematic illustration of the optimized procedure leading to the photo-imprinting of a channel waveguide (**a**) followed by that of diffraction gratings (for a double-grating architecture) (**b**). In (**b**), the view has been rotated by 90° in the horizontal plane.

The whole protocol adopted for the elaboration of our diffraction grating/channel waveguide fluorescent architecture is schematized in Figure 2, which also summarizes the main optimized experimental parameters, which are discussed in Figure 2.

**Figure 3:**
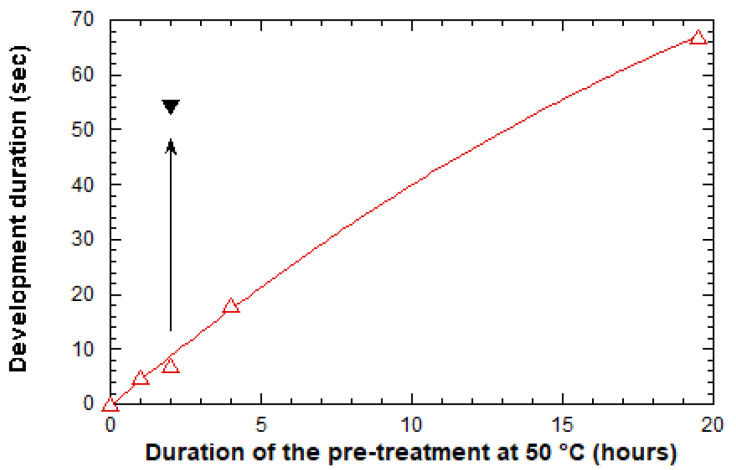
Influence of the pre-treatment duration at 50 °C on the grating development duration (red symbols). The black symbol accounts for a pre-treatment at 50 °C for 2 h, followed by a subsequent one at 110 °C for 10 min.

**Figure 4:**
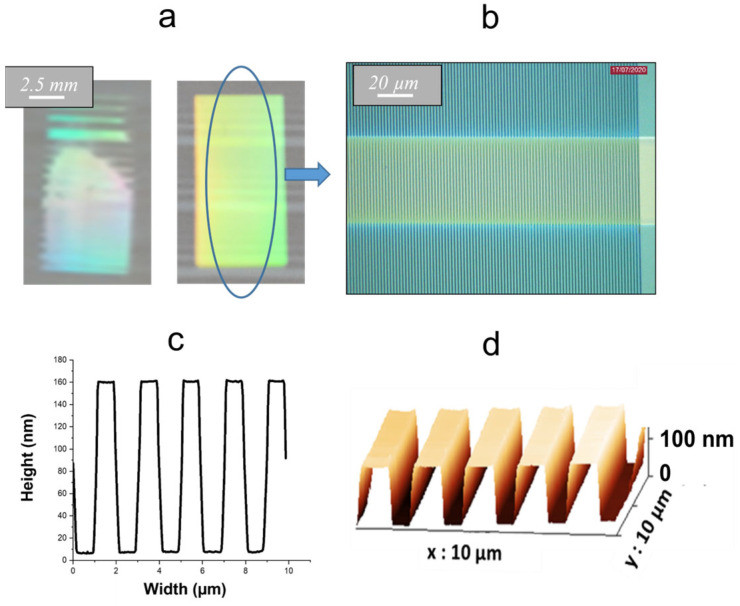
Macroscopic views of heterogeneous diffraction effects arising from our previous experimental procedure (left) and of uniform ones arising from the newly optimized procedure (right) (**a**), micrograph of a diffraction grating imprinted on a channel waveguide, according to the new procedure (**b**), and 2D (**c**) and 3D (**d**) AFM profiles of a resulting typical grating.

Channel waveguides derived from a photosensitive Ti-BzAc resist have been elaborated according to the procedure detailed in our previous paper [21] and recalled in Section 3. These experimental conditions lead to reproducible waveguides with a width at mid-height that fairly respects the expected width imposed by the transparent stripes of the lithography mask, and a trapezoidal profile with edge slopes of around 45°. These slopes have been attributed to diffraction effects on the edges of the transparent stripes during insolation through the mask. If the elaboration of the channel waveguides do not require further optimization, according to our previous work, it is not the same when considering diffraction gratings derived from the selective insolation of the Ti–BzAc resist. Their post-insolation development in ethanol requires an essential experimenter control that determines the visual apparition of diffraction effects and the time in which to stop development by rinsing the sample in water. However, contrary to the channel waveguides, this development is rendered extremely critical owing to the very weak dimensionalities of diffraction gratings (width/pitch of 1/2 µm). Accordingly, while some conformal gratings could sometimes be obtained, most importantly, they lacked height and width uniformity and reproducibility. It is illustrated by the micrograph of Figure 4a (left) showing very heterogeneous diffraction effects. This diffraction heterogeneity was associated with grating heights, measured by AFM, randomly varying over the whole photo-imprinted area (5 *×* 10 mm^2^) from 0 nm (no observed diffraction) to 160 nm. Several experimental tests were more recently performed to solve these problems. We finally came to suspect that the Ti–BzAc photoresist film experiences drying issues over the pre- and post-treatment involved in our experimental procedure. It is well-known that drying critically influences the chemical thickness uniformity of sol-gel deposited thin films [25]. During solvent evaporation and liquid film drying, the solvent is preferentially evaporated from the film surface. It causes a pressure gradient between the surface and deep layers of the film associated with a stiff–solid network at the surface, i.e., some kind of ‘surface crust’, while deep layers remain in a viscous liquid state. Derived tensile stresses often promote cracking of the film over drying when its thickness exceeds a certain threshold value, and this effect is all the more pronounced as the solvent evaporation proceeds rapidly. In our case, the deposited films were thin enough to overcome cracking issues, but we concluded that the formation of a surface crust during drying (pre- and post-treatment at 110 °C) could be at the origin of a critical and heterogeneous development of the diffraction gratings. Accordingly, we observed that this development proceeded through a first stage of a few seconds where no diffraction effects were visually observed, followed by an extremely rapid apparition (~1 s) of these effects, i.e., a fast and critical development of diffraction gratings. It was thus inferred that the first stage was associated with the duration necessary to firstly dilute the ‘chemically stable’ crust surface, while the second stage described the quasi-instantaneous dilution of chemically unstable liquid state-deep layers of the photoresist thin film. Based on these considerations, we have tested pre- and post-treatments at a weaker temperature (vs. 110 °C) in order to decrease the solvent evaporation kinetics and enhance the chemical thickness uniformity of the resist thin film. These features were expected to increase the development duration and thus reduce its criticality, as discussed below and illustrated in Figure 3 and Figure 4.

During the first attempts, we tested a reduction of the post-treatment temperature. These assays showed that a post-treatment at 50 °C for 1 h enabled to increase the development duration and decreased the criticality of this step, but the derived diffraction gratings still suffered from uniformity and reproducibility issues. During subsequent attempts, we kept these post-treatment’s at a constant (1 h at 50 °C), and we tested a reduction in the pre-treatment temperature, as illustrated in Figure 3. This figure shows that, for a sufficiently long pre-treatment at 50 °C, the development duration can be considerably increased, rendering this step much less critical. It thus allowed the elaboration of reproducible and uniform gratings in a 5 × 10 mm^2^ area. However, a development of sufficient duration (~1 min) required a long pre-treatment at 50 °C (~20 h), which overall, yielded a time-consuming experimental protocol. For this reason, we tested a dual pre-treatment by firstly performing a pre-treatment step of 2 h at 50 °C, followed by a second step of 10 min at 110 °C. As illustrated in Figure 3, this new pre-treatment led to a long development duration, involving an experimental procedure of reasonable duration, and once again yielding reproducible and uniform gratings, as depicted by the diffraction effects illustrated in Figure 4a (right) and the micrograph in Figure 4b. AFM profiles illustrated in Figure 4c,d show that derived gratings typically exhibit a height around 160 nm, a width at mid-height that fairly respects the expected value imposed by the transparent stripes of the lithography mask (1 µm), and as for channel waveguides, a trapezoidal profile with edge slopes around 45°. At the nanometric scale, these profiles also suggest a similarly good smoothness at the surface and on the walls of the gratings. Accordingly, analyses of 3D AFM profiles depicted a weak RMS roughness of 7.5 and 10 nm on the tops and on the walls of the grating, respectively. Finally, statistical AFM studies showed that the error on the grating height was around +/− 5% on the whole imprinted 5 × 10 mm^2^ area, and a similar error was deduced from measurements on different elaborated samples, which illustrates the grating uniformity and reproducibility. 

According to these studies, the channel waveguide/diffraction grating architectures were definitively elaborated using the following two-step procedure, schematically illustrated in Figure 2. Channel waveguides were elaborated from the first step, as described in the later Section 3, using pre- and post-treatments at 110 °C. In the second step, diffraction gratings were obtained through: (i) new spin-coating deposition of the sol-gel Ti–BzAc photoresist; (ii) dual pre-treatment (2 h at 50 °C followed by 10 min at 110 °C); (iii) selective insolation through a lithography mask for 10 min; (iv) post-treatment at 50 °C for 1 h; (v) development in ethanol and subsequent rinsing in water; and (vi) final heat-treatment at 110 °C for two hours. It should, however, be noticed that this new procedure leading to optimized gratings still required an essential experimenter control, since variations of climatic conditions in the experimental room (temperature, relative humidity) could significantly influence the resist chemical stability, and thus, the development duration.

#### 2.2.2. Complementary Optimization and Double Grating Architecture Elaboration

In subsequent studies, we tested the possibility to vary the height of diffraction gratings arising from our new procedure. In pioneering studies that are now commonly used as reference work, Bornside et al. divided the sol-gel deposition of thin films by spin-coating into four stages: Sol deposition on the immobilized substrate, spin-up (rapid acceleration of the substrate rotation), spin-off (rotation of the substrate at constant speed), and solvent evaporation/sol-gel transformation after substrate immobilization [26]. In this description, the amount of deposited material principally depends on the spin-off stage where, under centrifugal force driving, the liquid film thinners and liquid excess leaves the substrate as droplets. Thus, for the provided sol and rotation speed (fixed here at 3000 rpm), the resulting film thickness is expected to decrease when increasing the spin-off stage duration. Accordingly, ellipsometric measurements performed on full plate Ti–BzAc resist films, deposited on silicon substrates using the previously described two-step procedure, but without using a mask during insolation, showed that the resist thickness increased from ~160 to ~250 nm when decreasing the spin-off duration from 30 to 1 s, as illustrated in Table 1; for both considered durations, the height of derived channel waveguides and diffraction gratings closely matches the resist thickness. Interestingly, the resist refractive index exhibits a similar value around 1.75 (determined at the model wavelength of 633 nm) for both durations. For an identical film chemical composition, this index is expected to decrease when the film porosity increases. The fact that it does not vary with the rotation duration therefore shows that the enhanced thickness resulting from a shorter duration is not due to a porosity increase but only arises from an enhanced amount of deposited matter. Since optical simulations indicated that a 250 nm grating height could enhance light coupling with a channel waveguide, the rotation duration was definitely fixed at 1 s. 

**Figure 5:**
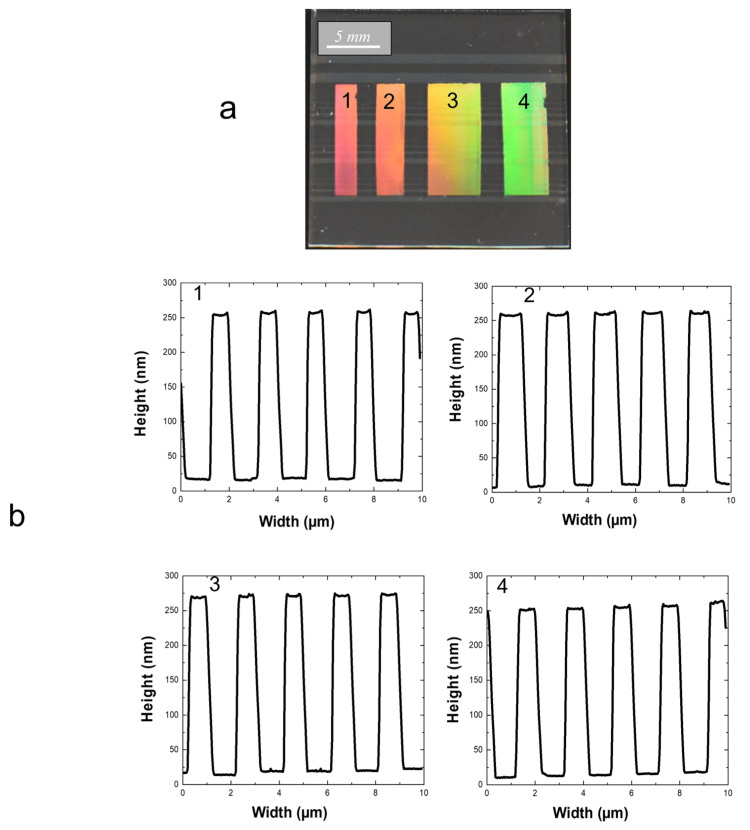
Macroscopic view (**a**) of diffracting stripes imprinted on a 1.2 × 2 cm^2^ area and separated by stripes constituted of denuded channel waveguides and (**b**) typical 2D AFM profiles of gratings constituting the different diffracting stripes indexed in (**a**).

Previously optimized experimental parameters enabled us to consider the elaboration of a complete architecture constituted of channel waveguides endowed with diffraction gratings at their input and output. For this purpose, in the second step of our procedure leading to diffraction gratings, we used an appropriate mask that comprised stripes constituted of transparent gratings with a width/pitch of 1/2 µm, and where these stripes were separated by chromium-coated ones. The mask was designed to imprint diffraction gratings of various lengths separated by bare-channel waveguides, as shown in Figure 5, for diffraction gratings imprinted in a 1.2 × 2 cm^2^ area. The macroscopic view of this figure illustrates four stripes that exhibit uniform diffraction effects. They are separated by stripes where no diffraction can be observed, i.e., where underneath-channel waveguides are totally denuded. The different diffracting stripes have been probed by AFM. Figure 5 illustrates that, in the whole considered area, the gratings exhibit a mean height of 250 nm lying within a small error range of +/−3%. This feature indicates, in particular, that decreasing the spinning duration from 30 to 1 s did not alter the uniformity of sol-gel thin films and derived gratings. Furthermore, the grating width at mid-height fairly respects the expected value of ~1 µm, even if the error range for this value is somewhat larger than that deduced for the grating height. Globally, these results definitely prove the robustness of our new optimized sol-gel procedure that enables the imprinting of uniform gratings on large areas and allows the well-controlled elaboration of a double grating architecture. In the next section, we show how such optimization is now compatible with accurate assessments based on fluorescence measurements in guided configuration.

### 2.3. Double Grating Architecture Assessments

#### 2.3.1. Preliminary Considerations

**Figure 6:**
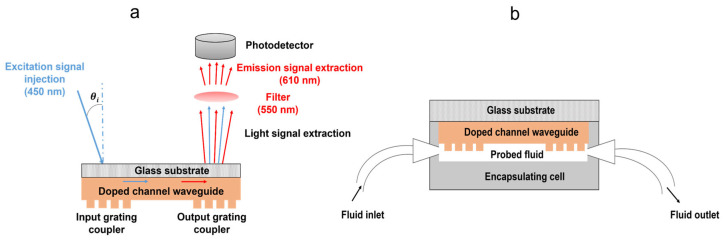
Illustration of fluorescence measurements performed in guided configuration with a double grating architecture (**a**) and schematic representation of the configuration intended for future measurements in liquid medium after integration of the architecture in a micro-fluidic cell (**b**).

Before assessing our double grating sol-gel architecture by experimental measurements, it is necessary to address preliminary considerations. In the following, fluorescence measurements will be performed in a configuration where the excitation (emission) signal is injected in (extracted from) the back side of the substrate, i.e., the bare face not coated with the architecture (Figure 6a). This configuration was adopted in the view of future measurements in the liquid medium after integration of the architecture in a micro-fluidic cell, as schematically illustrated in Figure 6b. It therefore differs from those which were adopted for simulations, since these were performed by considering light coupling with a guided mode occurring from a plane-wave directly impinging on, or radiated, from the grating at the air-grating interface. Thus, contrarily to the configuration adopted for simulations, it is necessary in our experimental conditions to consider optical diffraction at the different interfaces (air–glass, glass–waveguide, waveguide–grating), which is expected to modify the light incidence according to the travelled media-refractive indices and to the Snell–Descartes equation (n_1_sinθ_1_ = n_2_sinθ_2_). However, our experimental conditions imply that the gratings imprinted at the architecture surface are surrounded by an external medium, which in this work, is air. Accordingly, a simple calculation based on the Snell–Descartes equation shows that the simulated configuration and the experimental one are both described by sinθ_i_ = n_g_sinθ_g,_ where θ_i_ and θ_g_ account for the light-injection angle in air and that of light travelling the grating, respectively, and n_g_ accounts for the grating-refractive index. It means that, for a uniaxial excitation beam at a wavelength around 460 nm, the 55° injection angle derived from simulations for an optimal grating/waveguide coupling should also be valid in our experimental configuration. 

**Figure 7:**
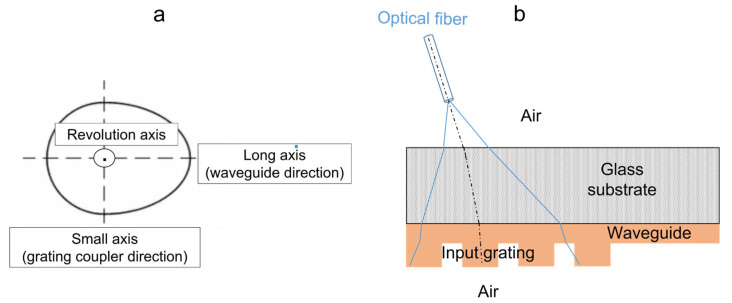
Illustration of the ovoidal beam illuminating the architecture surface (**a**) and schematic cross-section view showing its long-axis evolution when light travels through the different media, constituting the architecture in our experimental conditions (**b**).

There is, however, an important difference between both considered configurations, which is related to the numerical aperture (NA) of the injection fiber (0.12). According to this NA, the injected light signal is not uniaxial, as supposed in our simulations, but it emerges from the fiber as a conical beam with an angle of +/− 7° around the fiber axis. Moreover, according to the non-vertical incidence adopted for the excitation signal, the beam illuminating the sample surface presents an ovoidal profile, which is schematized in Figure 7a. The dimensions of this beam (small and long axes), in turn, depend on refraction effects when light travels through the different media. Accordingly, while these dimensions are quite reduced in the configuration used for simulations, i.e., a few tens of micrometers depending on the fiber-sample vertical distance, they considerably increase after light travels through the glass substrate and waveguide thicknesses in our experimental conditions (Figure 7b). For instance, a simple calculation (not detailed here) based on the Fresnel law and considering the fiber NA showed that, in the experimental configuration, the long axis (along the channel waveguide axis) of the ovoidal beam illuminating the input grating of our architecture, exhibits a length around 200 µm, 230 µm and 550 µm for an incidence angle in air of 55°, 70° and 80° vs. the vertical direction, respectively. Obviously, according to these dimensions, the simulations assuming a uniaxial light beam cannot perfectly account for channel waveguide/diffraction grating coupling in our experimental conditions. However, it is inferred that any discrepancy between the simulated configuration and the experimental one is not that important, since in both cases the beam emerging from the injection fiber is expected to present a Gaussian-like intensity profile, where the maximal intensity is aligned on the fiber axis, which is analyzed hereafter. 

#### 2.3.2. First Experimental Assessments

**Figure 8:**
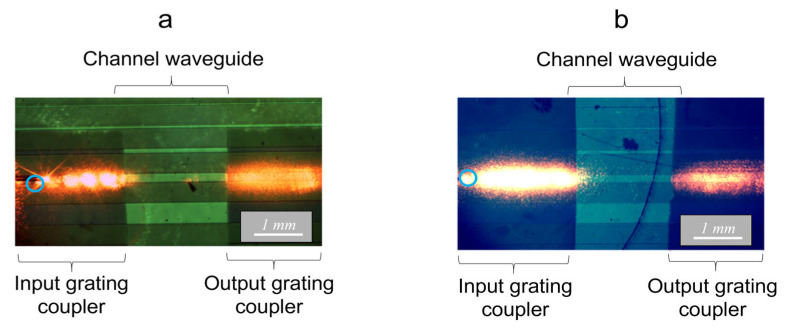
Micrographs illustrating light propagating in a double grating architecture after light injection (635 nm, 55° vs. the vertical direction) from the coated face (**a**) and bare face of the substrate (**b**). The circles depict the vertical to the fiber extremity.

Experimental assessments were performed on architectures constituted of gratings with a length of 5 mm and a width/pitch of 1/2 µm, imprinted at the input and output of a channel waveguide of 200 µm width. The distance between both gratings, i.e., the length of the bare waveguide, was fixed at 2 mm. Figure 8 firstly illustrates observations made under illumination at 635 nm (a wavelength value close to the Rudpp emission) on an architecture including a waveguide not doped with Rudpp, i.e., where no fluorescence emission is considered. These observations were performed with a fiber incidence arbitrarily fixed at 55° (as this part is only devoted to qualitative visualizations, we did not try to optimize the grating/waveguide coupling), where light directly illuminated the input grating, as supposed to our simulation configuration (Figure 8a), or illuminated this grating after light traveled through the glass substrate and waveguide thickness, as considered in our experimental configuration (Figure 8b). In both experimental configurations, the fiber extremity was positioned at the vertical of the input grating, at around 2 mm from the interface with the bare waveguide, and the fiber-sample vertical distance was fixed at 30 µm. These conditions were fixed according to preliminary experiments (not illustrated here). 

Figure 8a illustrates an intense longitudinal light beam nearly extending from the vertical to the fiber extremity position up to the grating/waveguide interface. This beam depicts light coupled in the channel waveguide and propagating in its core below the grating. A part of the signal is expected to propagate in the core of the bare waveguide toward the output grating. Another part of the signal is expected to be extracted (decoupled) through the grating toward the upper surrounding medium, which allows its visualization. Some separated spots of enhanced light intensity are also observed, which probably correspond to light preferentially extracted according to specific decoupling orders. It is also noticed that the width of the propagating light signal is greater than the 200 µm channel waveguide. While diffraction effects promoted by the grating preferentially suppose signal coupling and propagation in the waveguide axis, light can also be planarly diffracted in various directions according to the ovoidal illumination profile, which may explain that the observed signal is not rigorously confined within the channel waveguide width. Figure 8a also illustrates similar light features at the vertical of the output grating, i.e., an intense, elongated and quite-large signal emerging from the bare waveguide and propagating below the grating, through which it is extracted toward the upper surrounding medium and subsequently visualized. In contrast, no signal is observed between both input and output gratings, showing that the propagating light signal is efficiently confined in the bare channel waveguide and eventually in the glass substrate. 

The micrograph of Figure 8b illustrates quite similar light behaviors, i.e., the visualization of an intense, elongated and quite-large signal at the vertical of the input and output gratings, and the absence of optical losses when light propagates along the waveguide core. This globally depicts that, for a same incidence of the injected signal, comparable light coupling features arise from the simulated configuration (Figure 8a), and that the subsequent fluorescence measurements in guided configuration utilize the double grating architecture (Figure 6 and Figure 8b). However, some differences must be noticed between both configurations. As illustrated in Figure 8b, at the vertical of the input grating, the elongated light beam appears to be larger and longer compared to what is illustrated in Figure 8a. Beside the previously discussed planar diffraction effects, this feature also presumably arises from the enhanced dimensions of the ovoidal beam illuminating the grating after light travelling through the substrate and waveguide thicknesses. The light extracted toward the external medium also appears much more intense, as depicted by the dominating bright aspect of the elongated beam, and separated light spots are no longer observed. These features probably depict that, since after travelling the different media, the conical light beam illuminates the input grating within an enhanced angular range; it allows a more efficient extraction of light toward the upper external medium according to an enhanced number of decoupling orders. Thus, a reduced fraction of light is expected to be injected in the bare-waveguide axis and propagate toward the output grating. Accordingly, Figure 8b illustrates that the intensity of light extracted at the vertical of this grating is weaker than that depicted in Figure 8a. These differences do not, however, call into question an experimental configuration where excitation and emission signals are, respectively, injected and extracted from the backside of the substrate, as presented next. 

#### 2.3.3. Fluorescence Measurements in Guided Configuration

**Figure 9:**
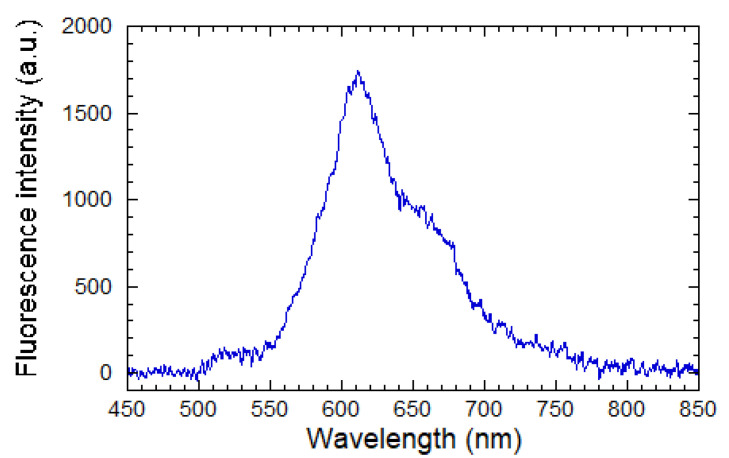
Emission spectrum of a Ti–BzAc resist film doped with Rudpp under excitation at 450 nm (reproduced from [21]).

The emission spectrum of a Ti–BzAc resist film doped with Rudpp and measured in transmission on a full plate sample (not in waveguiding configuration), is illustrated in Figure 9 according to our previous paper [21]. This spectrum particularly illustrates that the 450 nm wavelength adopted in the following is suitably adapted to excite the Rudpp fluorophore encapsulated in the Ti–BzAc host marix. 

**Figure 10:**
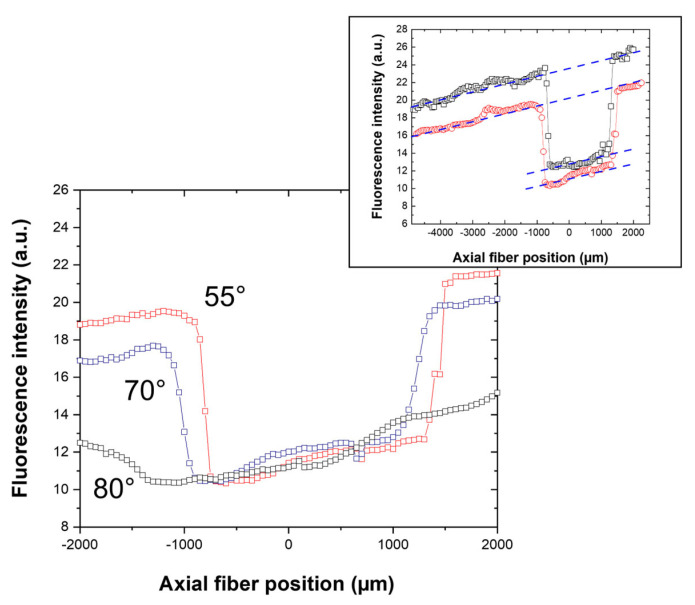
Influence of the excitation fiber (450 nm) axial displacement on the fluorescence intensity, measured with a photodiode positioned at the vertical of the output grating for various excitation incidence angles. The insert shows similar features for two additional samples excited with a 55° incidence. The fiber position has been swept from the vertical to the output grating (**right**) up to the vertical to the input grating (**left**) and, in the abscise axis, the zero value corresponds to the fiber output positioned at the vertical of the input grating—channel waveguide interface.

Accordingly, fluorescence measurements were then performed in guided configuration on the same architecture, as that which is previously described and schematically illustrated in Figure 6a, using a photodiode, a Rudpp-doped channel waveguide, and an excitation wavelength of 450 nm. As before, the fiber-sample vertical distance was fixed at 30 µm and, to deepen the analysis of light coupling and propagation features by fluorescence measurements, we chose to scan the position of the excitation fiber along the waveguide axis from the vertical to the output grating to that of the input one. Fluorescence measurements were achieved for fiber incidence angles of 55, 70 and 80° and fiber scanning was performed with a step of 50 µm and a light exposure duration of 10 s for each measurement. The fluorescence intensity was measured using a photodiode of 3.6 × 3.6 mm^2^ surface vertically positioned above the output grating. Such a large surface allowed an optimal detection of the fluorescence signal emitted in the various directions suggested by the simulations. The photodiode-glass substrate vertical distance was fixed at ~3 mm, which appeared as a suitable trade-off between an optimal collection of the emission signal and sufficient spacing, enabling to perform fiber scanning on the whole intended-axial distance. The results are illustrated in Figure 10, which globally illustrates similar intensity trends for the three inspected angles, i.e., two intensity “plateaus” separated by a “well” of weaker intensity. It is inferred that these trends describe diffraction grating-channel waveguide light coupling. This coupling should be effective when the ovoidal excitation beam is positioned at the vertical of the input or output grating, which leads both to the axial propagation of the excitation and emission signals. In contrast, when the ovoidal beam is positioned between the gratings, at the vertical of the bare-channel waveguide, no excitation signal coupling occurs with the waveguide. In that case, excitation of some Rudpp molecules happens without involving a guided propagation, and only the resulting emission signal is axially propagated. Intensity differences between the plateaus and wells, which are illustrated in Figure 10, depict the light coupling efficiency when diffraction gratings are illuminated by the excitation beam. This efficiency is observed to be very weak for an incidence of 80° and to be optimal for an incidence of 55°, which matches the optimal angle value deduced from the simulations. It means that, despite strong differences between configurations adopted in our experimental conditions and for the simulations, the latter seem to fairly describe light coupling features in the experimental conditions. However, it will be necessary in further optimizations to better define experimentally what the optimal incidence around the 55° value is. The good repeatability of experimental measurements is also illustrated in the insert of Figure 10, as depicted by the study of two additional samples using a fiber incidence of 55°. Only a slight shift in the intensity level of the plateaus and wells is observed (see doted lines in the insert), which may be due to small modifications in the experimental configuration when changing the sample; for instance, the longitudinal or vertical positioning of the excitation fiber and/or photodiode relative to the sample.

Figure 10 also shows that the position and profile of intensity wells are strongly influenced by the incidence angle of the excitation beam. In our description, the well walls account for the position of the fiber when this beam crosses the interface between the input or output grating and the bare-channel waveguide. However, the ovoidal excitation beam does not abruptly cross both interfaces according to its length, which causes a slope of the intensity well walls. As previously mentioned, in our experimental conditions, increasing the incidence angle yields a significant enhancement in the ovoidal beam length (200 µm, 230 µm and 550 µm at 55°, 70°, and 80°, respectively). Accordingly, well’s slopes illustrated in Figure 10 are observed to decrease, i.e., to depart from the vertical direction, as the incident beam angle increases from 55 to 80°. On the one hand, this decrease is particularly marked for an incidence angle of 80° owing to the important corresponding beam length. On the other hand, for incidences of 55° and 70°, Figure 10 depicts rather similar well profiles with walls closer to the vertical, the most ‘vertical’ profile being observed for a 55° incidence. These features are in agreement with the ovoidal beam length deduced for both incidences. Furthermore, Figure 10 illustrates intensity wells all the more shifted toward the input grating as the incidence angle increases. In our description, the tops of the left well walls (of the right well walls) depict a fiber-position limit where the input grating-channel waveguide light coupling ceases to be optimal (where the channel waveguide-output grating light coupling starts to be optimal). Thus, owing to an enhanced beam length, an optimal light coupling between the channel waveguide and both gratings is expected to occur for a fiber position displaced on the left, far from the grating-waveguide interface, when the excitation signal incidence increases, which causes a well shift toward this interface. All these analyses support the intensity plateaus and wells illustrated in Figure 10, which depict light coupling features with the channel waveguide when diffraction gratings are illuminated by the excitation beam. Additional data depicted in this figure are analyzed hereafter. 

#### 2.3.4. Complementary Analyses and Fluorescence Measurements

**Figure 11:**
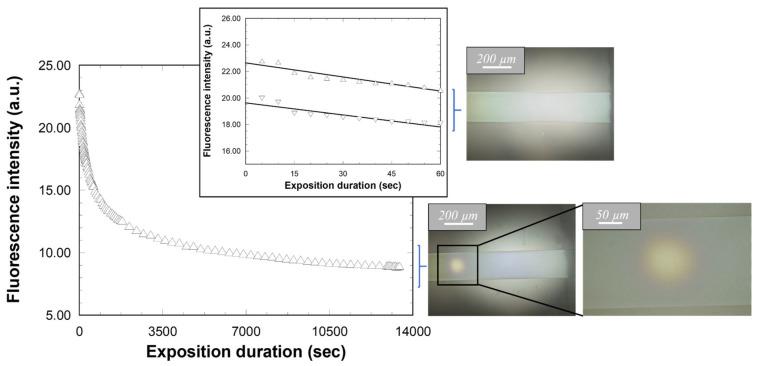
Influence of the excitation duration (450 nm) on the fluorescence intensity measured for a fixed-fiber position. The insert shows same variations for two different samples over a short exposure duration. The micrographs illustrate the sample aspect after an exposition of 60 s (**top**) and 4 h (**bottom**).

Figure 10 illustrates that, for the different inspected angles, intensity levels in the plateaus and wells are modulated by a similarly weak and somewhat linear slope, where the intensity slightly decreases when the excitation fiber is displaced from the output to the input grating. For instance, for the plateaus observed with a 55° incidence, the intensity decrease is about 3%/mm. This slope can partly be attributed to some optical losses when the emission signal propagates in the waveguide core axis. For instance, since fluorescence is isotropically emitted in the space, part of the signal does not remain confined in the waveguide core, but is instead radiated in the surrounding media, particularly in the glass substrate. However, the signal radiated in the substrate is not entirely lost, since it is confined, and propagates within the glass thickness, i.e., the substrate acts as a planar waveguide. The propagated signal can then be coupled with the output grating and collected by the photodiode, which should significantly reduce the effects of propagation losses. Another feature can also participate in the observed slope, namely an alteration of the Rudpp emission caused by its photobleaching. This phenomenon is due to a photochemical reaction of the fluorophore under light excitation, which induces the formation of photoproducts that are no longer emissive [27]. It is all the more marked as the excitation dose (power and/or duration) or the oxygen concentration increases, and it is also strongly influenced by the liquid or solid Rudpp encapsulating medium. Thus, new fluorescence measurements were performed to assess the influence of this phenomenon in our experimental conditions, as illustrated in Figure 11. For these measurements, the excitation fiber incidence and vertical position were once again fixed at 55° and 30 µm, respectively, and the fiber output was positioned at the vertical of the input grating with a fixed distance of 1500 µm from the interface between this grating and the bare channel waveguide, i.e., on the left intensity plateau illustrated in Figure 10a. 

Figure 11 shows that the fluorescence intensity follows some kind of exponential decrease over excitation at 450 nm. Compared to the initially measured value, the intensity reached after excitation for 1 h is divided by a factor of 2, and it undergoes further decrease of about 20% when the sample is subsequently excited for 3 h. After 4 h of light exposure, the micrographs of Figure 11 (bottom) illustrate a localized modification of the sample visual aspect in a circular area, which is located at the vertical of the excitation fiber output. On the one hand, these features depict the effects of photobleaching over a prolongated light exposition. On the other hand, the insert of Figure 11 shows that the intensity decrease remains limited to only ~10% after 1 min of excitation. This trend has been reproduced on two different samples, which provides new evidence of the repeatability of our experiments. Only a slight intensity shift is observed between both measurement series, which can once again be attributed to small modifications in the experimental device when changing the sample. However, the micrograph of Figure 11 (top) shows that such a short light exposure does not induce any modification of the sample visual aspect. These features demonstrate that, for an excitation of short duration, photobleaching takes place in a very limited extent, which has a practical consequence on the analysis of Figure 9. When considering experimental conditions adopted for data illustrated in this figure (a step of 50 µm and a light exposure duration of 10 s for each measurement) and the 200 µm beam length for a fiber incidence of 55°, it appears that the fluorophore is directly exposed to the excitation beam, i.e., in conditions susceptible to promote photobleaching, for only 30–40 s. According to the insert of Figure 11, this short illumination is expected to induce a very limited photobleaching. Consequently, these analyses enable us to describe slopes, as illustrated in Figure 10, showing that the experimental measurements are affected by very reduced propagation losses and photobleaching features. From a more general point of view, all these studies demonstrate that, thanks to an optimized elaboration procedure leading to a reproducible and uniform sol-gel architecture, the latter allows us to perform reliable fluorescence measurements in guided configuration. Furthermore, this has been achieved in a configuration compatible with integration in a microfluidic platform adapted to measurements in the liquid medium. This last feature has enabled us to undertake our objective in this work. 

## 3. Materials and Methods

### 3.1. Sol-Gel Processing

A transparent and high-refractive index (n~1.85 and n~1.75 at the Rudpp excitation (460 nm) and emission wavelength (610 nm), respectively [20]), TiO_2_-based guiding layer was deposited using a low temperature sol-gel procedure that has been fully optimized and detailed in our previous paper [28], according to pioneering works performed by Tohge et al. on UV lithographic patterning of TiO_2_–BzAc-based materials [29]. A low temperature process constitutes an essential aspect of consideration, in order to: (i) preserve the integrity and photo-activity of Rudpp that will be then encapsulated in the guiding layer, and (ii) preserve the photosensitivity of the TiO_2_-based photoresist used for further photo-imprinting. Briefly, a sol was prepared from tetraisopropylorthotitanate (TIPT) and Benzoylacetone (BzAc) diluted in a methanol–butanol–deionized water–hydrochloric acid mixture. The BzAc–TIPT molar ratio and TIPT concentration were fixed at 0.6 and 0.5 M, respectively. To deposit waveguiding layers doped with the fluorophore, a stock solution with a Rudpp concentration of 12.5 mM was prepared in absolute ethanol and a suitable volume of this solution was then added to the Ti-BzAc sol to fix the final Rudpp concentration at 1 mM. The sol was then deposited by spin-coating at 3000 rpm on 2.5 × 2.5 cm^2^ soda-lime glass substrates (refractive index of ~1.52 in the range of wavelengths considered in this work). The obtained liquid films underwent a sol-gel transformation at room temperature and were subsequently heat-treated at 110 °C for 10 min, leading to doped or non-doped Ti–BzAc xerogel thin films. As previously detailed, the Ti–BzAc complex plays a key role in this sol-gel formulation for the elaboration of a micro-structured architecture [18,19]. On the one hand, such a complexation reduces the sol-gel reactivity of TIPT, so that Ti–BzAc xerogel films, deposited at room temperature and heat-treated at 110 °C, are chemically unstable and can easily be leached through simple washing with alcohol. On the other hand, BzAc is a photosensitive reagent that undergoes partial photolytic decomposition of its chelate ring when exposed to UVA light. This decomposition leads to alcohol insoluble species (carbonates, carboxylates) that promote the chemical stabilization of the Ti–BzAc xerogel film. These features promote the formation of a negative resist where, after selective insolation and appropriate washing, areas exposed to UVA lights remain intact, while non-exposed areas are completely removed from the substrate. The photoresist can therefore be imprinted through a single photolithography step, which has been exploited in this work to elaborate channel waveguide/diffraction grating architectures. 

### 3.2. Micro-Structured Architecture Elaboration

According to our previous work [21], a two-step deposition/insolation lithographic procedure has been implemented to elaborate such architectures. In the first step, a Ti–BzAc xerogel film doped with Rudpp, deposited by spin-coating and thermally pretreated at 110 °C for 10 min, was insolated at 365 nm for 40 min using a commercial device (UV-KUB from KLOE) set in contact mode through a chromium mask. This mask comprised linear UVA-transparent stripes of various widths covering the whole length (2.5 cm) of the insolated sample. A post-insolation heat-treatment was then performed at 110 °C for 8 min to enhance the photo-induced solubility contrasts between insolated or non-insolated areas. After that, the derived patterns were developed in absolute ethanol for a dozen of seconds, rinsed in deionized water to stop the development, and then gently dried with a nitrogen spray. This first step led to the elaboration of channel waveguides of various widths according to the dimensionalities of transparent stripes constituting the used mask. The samples were finally heat-treated at 110 °C for two hours to enhance the waveguide chemical stability. A similar pretreatment/insolation through a chromium mask (for 10 min)/post-treatment procedure was then repeated, after which, the sample was washed in ethanol to develop the diffraction grating (non-doped with Rudpp), elaborated at the waveguide surfaces and once again heat-treated at 110 °C for two hours. As explained in the Introduction, the period of the diffraction grating should ideally be on the order of the wavelengths of interest (around 460 and 610 nm in this work). However, owing to diffraction limits, well-resolved patterns of width which are inferior to 1 µm can hardly be obtained in our conditions using traditional photolithography with a chromium mask. Thus, for this second lithographic step, we used a mask comprising linear UVA-transparent stripes with a width/pitch of ½ µm. More detailed data on the elaboration of an architecture, which constituted channel waveguides endowed with diffracting gratings at their input and output, enabled the object of optimization studies that were described in Section 2, and the overall resulted protocol is illustrated in Figure 4. In this study, thin film architectures were also synthesized on silicon wafers to perform ellipsometric measurements on full-plate samples elaborated in the same conditions as those used for the channel waveguide/diffraction grating architecture elaboration. In that case, the previously described double pretreatment/insolation/post-treatment procedure was also used, except that no lithographic mask was employed over the insolation steps. 

### 3.3. Theoretical Analyses

Light coupling at various wavelengths *λ* between channel waveguides and diffraction gratings imprinted at their surface was assessed by theoretical analyses. Light propagating in a channel waveguide is characterized by a discrete number of Transverse Electric (TE)- and Transverse Magnetic (TM)-guided modes of effective index *n_eff_*. In the studied configuration, light can be injected in or extracted from the waveguide according to a finite number of diffraction angles, *θ_d_*, by means of grating couplers having an appropriate period *Λ*. Basically, light coupling with a guided mode is described by the so-called grating law [30]:

(1) $$n_{\sup}\sin\left(\theta_{d}\right)=n_{\mathrm{eff}}-\frac{q\lambda }{\Lambda }\;$$

where *n_sup_* is the refractive index of the superstrate and *q* is an integer that represents the different diffracted-coupling orders. Thus, Equation (1) enables to determine *θ_d_* angles where light coupling with the guided mode occurs from a plane-wave impinging on the grating, or where guided light is radiated through the grating toward the external medium. However, this equation does not take into account grating dimensionalities (height, width, profile, length) other than the period. Thus, optical simulations are necessary to derive more reliable insights in the light coupling efficiency. These simulations have been performed using AFMM (Aperiodic Fourier Modal Method), also known as RCWA (Rigorous Coupled Wave Analysis), modified with PMLs (Perfect Matching Layers) [31,32]. Implementation of the adopted model, which takes into account the dimensionalities of gratings constituting our architecture, has been detailed in our previous paper [21]. In the present work, the method has been used to simultaneously determine the diffraction angles, *θ_d_*, and their associated diffracted coupling orders, q, in a configuration where light is propagating in the waveguide core at various wavelengths in TE polarization mode. 

### 3.4. Characterization

The opto-geometrical properties of diffraction gratings and channel waveguides were quantified by Atomic Force Microscopy (AFM) with a Bruker Dimension Icon device operated in tapping mode, and using a Scanasyst-air triangular geometry tip mounted on a silicon–nitride cantilever. Large-scale uniformity of the samples was evaluated by optical microscopy using a Leica apparatus. The thickness and refractive index of full-plate Ti–BzAc xerogel films (without selective insolation) deposited on silicon wafers were measured for reference by spectroscopic ellipsometry using an AutoSE HORIBA Scientific device. To extract these values, spectral data acquired in the 300–700 nm wavelength range were fitted with the empirical Cauchy model, which is commonly employed for transparent materials. Fluorescence measurements in guided configuration were performed with a home-made bench, schematized in Figure 6a, and using a Thorlabs LP450-SF15 laser source emitting at 450 nm. The signal emitted by the laser source was injected from the back side of the substrate using a Thorlabs S405-XP fiber with a 4 µm diameter and a 0.12 numerical aperture, and a devoted set-up allowing precise angular and XYZ positioning of the fiber was employed. The excitation signal was transmitted through the substrate and channel waveguide thicknesses, and could then be coupled with a diffraction grating at the waveguide input for further guided propagation in the waveguide core axis. It enabled excitation of the Rudpp fluorophore along the waveguide axis and subsequent guided propagation of the emission signal, which was then radiated through an output grating toward the substrate. After transmission through this process, the emission signal was collected using a Thorlabs FDS100 Silicon Photodiode. A FEL0550 Thorlabs long-pass filter, with a 550 nm cutoff wavelength, was inserted between the sample and the photodiode to eliminate the excitation signal emerging from the substrate, thus allowing a selective detection of the Rudpp emission. More detailed data on these fluorescence measurements are provided in the ‘Results and discussion’ section. Punctual experiments were additionally performed using a Thorlabs S1FC635PM laser source emitting at 635 nm, whose details are also presented in this section.

## Figures and Tables

**Table 1:** Influence of the rotation duration at 3000 rpm on the thickness and refractive index at 633 nm of a full plate Ti–BzAc resist film and on the height of derived channel waveguides and diffraction gratings.

Rotation Duration (s)	Ti–BzAc Resist Thickness (nm)/Index	Waveguide Height (nm)	Grating Height (nm)
1	250/1.75	250	250
30	160/1.75	160	160

## Data Availability

Not applicable here.

## Sample Availability

Not available.

## References

[1] Oubaha M., Gorin A., McDonagh C., Duffy B., Copperwhite R. (2015). Development of a multianalyte optical sol-gel biosensor for medical diagnostic. Sens. Actuators.

[2] Liu L., Zhou X., Wilkinson J.S., Hua P., Song B., Shi H. (2017). Integrated optical waveguide-based fluorescent immunosensor for fast and sensitive detection of microcystin-LR in lakes: Optimization and Analysis. Sci. Rep..

[3] Walter J.-G., Alwis L.S.M., Roth B., Bremer K. (2020). All-optical planar waveguide-based biosensor chip designed for smartphone-assisted detection of vitamin D. Sensors.

[4] Azuelos P., Girault P., Lorrain N., Poffo L., Guendouz M., Thual M., Lemaitre J., Pirasteh P., Hardy I., Charrier J. (2017). High sensitivity optical sensor based on polymer materials and using the Vernier effect. Opt. Express.

[5] Hajj-Hassan M., Gonzalez T., Ghafar-Zadeh E., Djeghelian H., Chodavarapu V., Andrews M., Therriault D. (2008). Direct-dispense polymeric waveguides platform for optical chemical sensors. Sensors.

[6] Ram R.J., Lee K. Optical waveguides for microfluidic integration. Proceedings of the 2009 IEEE LEOS Annual Meeting Conference Proceedings.

[7] Alberti S., Jagerska J. (2021). Sol-gel thin film processing for integrated waveguide sensors. Front. Mater..

[8] Kazmierczak A., Butt M.A., Zieba M., Tyszkiewicz C., Karasinski P., Piramidowicz R. (2022). Towards the most convenient configuration of integrated photonic sensor for implementation in SiO_2_:TiO_2_ sol-gel derived waveguide film technology. Proc. SPIE.

[9] Righini G.C., Armellini C., Ferrari M., Carlotto A., Carpentiero A., Chiappini A., Chiasera A., Lukowiak A., Tran T.N.L., Varas S. (2023). Sol-Gel Photonic Glasses: From Material to Application. Materials.

[10] Le S.D., Delcourt E., Girault P., Guttierez-Arroyo A., Azuelos P., Lorrain N., Bodiou L., Poffo L., Goujon J.M., Dumeige Y. (2016). Study of optimized coupling based on micro-lensed fibers for fibers and photonic integrated circuits in the framework of telecommunication and sensing applications. Commun. Phys..

[11] Dai D., Tang Y., Bowers J.E. (2012). Mode conversion in tapered submicron silicon ridge optical waveguides. Opt. Express.

[12] Cardenas J., Poitras C.B., Luke K., Luo L.W., Morton P.A., Lipson M. (2014). High coupling efficiency etched facet tapers in silicon waveguides. IEEE Photonic Technol. Lett..

[13] Liu J., Raja A.S., Pfeiffer M.H.P., Herkommer C., Guo H., Zervas M., Geiselmann M., Kippenberg T. (2018). Double inverse nanotapers for efficient light coupling to integrated photonic devices. Opt. Lett..

[14] Taillaert D., Van Laere F., Ayre M., Bogaerts W., Van Thourhout D., Bienstman P., Baets R. (2006). Grating couplers for coupling between optical fibers. Jpn. J. Appl. Phys..

[15] Demeter A., Ruschin S. (2016). Back-reflecting interferometric sensor based on grating coupler on a planar waveguide. J. Opt..

[16] Kuswandi B., Nuriman, Huskens J., Verboom W. (2007). Optical sensing systems for microfluidic devices: A review. Anal. Chim. Acta.

[17] Lambeck P.V. (2006). Integrated optical sensors for the chemical domain. Meas. Sci. Technol..

[18] Mukundan H., Anderson A.S., Grace W.K., Grace K.M., Hartman N., Martinez J.S., Swanson B.I. (2009). Waveguide-based biosensors for pathogen detection. Sensors.

[19] Enami Y. (2017). Fabricating 90 nm resolution structures in sol-gel silica optical waveguides for biosensor applications. J. Sens..

[20] Bonnel M., Riassetto D., Morand A., Bucci D., Langlet M. (2019). Micro-structuration of a sol-gel architecture for channel waveguide/diffraction grating coupling. Opt. Mater..

[21] Bonnel M., Marzouk I., Riassetto D., Morand A., Bucci D., Langlet M. (2022). Setting up and assessing a new micro-structured waveguiding fluorescent architecture on glass entirely elaborated by sol–gel processing. Materials.

[22] Demuth C., Varonier J., Jossen V., Eibl R., Eibl D. (2016). Novel probes for pH and dissolved oxygen measurements in cultivations from millilitre to benchtop scale. Appl. Microbiol. Biotechnol..

[23] Barczak M., McDonagh C., Wencel D. (2016). Micro- and nanostructured sol-gel based materials for optical chemical sensing. Microchim. Acta.

[24] Mills A., Graham A., O’Rourke C. (2014). A novel titania sol-gel derived film for luminescence-based oxygen sensing. Sens. Actuators.

[25] Brinker C.J., Scherer G.W. (1990). Sol-Gel Science—The Physics and Chemistry of Sol-Gel Processing.

[26] Bornside D.E., Macosko C.W., Scriven L.E. (1987). Modeling of spin coating. J. Imaging Technol..

[27] Hartmann P., Leiner M.J.P., Kohlbacher P. (1998). Photobleaching of a ruthenium complex in polymers used for oxygen optodes and its inhibition by singlet oxygen quenchers. Sens. Actuators.

[28] Briche S., Tebby Z., Riassetto D., Messaoud M., Gamet E., Pernot E., Roussel H., Dellea O., Jourlin Y., Langlet M. (2011). New insights in photo-patterned sol-gel derived TiO_2_ films. J. Mater. Sci..

[29] Tohge N., Shinmou K., Minami T. (1994). Effects of UV-irradiation on the formation of oxide thin films from chemically modified metal-alkoxides. J. Sol.-Gel Sci. Technol..

[30] Yariv A., Yeh P. (2006). Photonics: Optical Electronic in Modern Communications.

[31] Hugonin J.P., Lalanne P. (2005). Perfectly matched layers as nonlinear coordinate transforms: A generalized formalization. J. Opt. Soc. Am. A.

[32] Bucci D., Martin B., Morand A. (2012). Application of the three-dimensional aperiodic Fourier modal method using arc elements in curvilinear coordinates. J. Opt. Soc. Am. A.

